# A Hybrid Framework for Lung Cancer Classification

**DOI:** 10.3390/electronics1010000

**Published:** 2022-05-18

**Authors:** Zeyu Ren, Yudong Zhang, Shuihua Wang

**Affiliations:** School of Computing and Mathematical Sciences, University of Leicester, Leicester LE1 7RH, UK

**Keywords:** deep learning, generative adversarial networks, image classification, neural network, transfer learning

## Abstract

Cancer is the second leading cause of death worldwide, and the death rate of lung cancer is much higher than other types of cancers. In recent years, numerous novel computer-aided diagnostic techniques with deep learning have been designed to detect lung cancer in early stages. However, deep learning models are easy to overfit, and the overfitting problem always causes lower performance. To solve this problem of lung cancer classification tasks, we proposed a hybrid framework called LCGANT. Specifically, our framework contains two main parts. The first part is a lung cancer deep convolutional GAN (LCGAN) to generate synthetic lung cancer images. The second part is a regularization enhanced transfer learning model called VGG-DF to classify lung cancer images into three classes. Our framework achieves a result of 99.84% ± 0.156% (accuracy), 99.84% ± 0.153% (precision), 99.84% ± 0.156% (sensitivity), and 99.84% ± 0.156% (F1-score). The result reaches the highest performance of the dataset for the lung cancer classification task. The proposed framework resolves the overfitting problem for lung cancer classification tasks, and it achieves better performance than other state-of-the-art methods.

## 1 Introduction

Cancer leads to 10 million deaths per year and relates to a series of diseases where the abnormal cells randomly mutate inside the human body [1]. When organs generate abnormal cells, these cells are spread throughout the body and eventually cause death. The most commonly affected organs are lungs, liver, stomach, colon, skin, prostate, and brain. There are various causes of cancer. The primary reasons for cancer range from daily behaviour such as smoking, high body mass index, or alcohol intake to exposure to physical carcinogens like radiation, ultraviolet, and electromagnetic fields. Canceration results from the interaction between a person’s genetic factors and three types of external factors including chemical carcinogens, biological carcinogens, and genetic carcinogens [1]. Among all the types of cancers, lung cancer is the leading cause of death globally, and the number of deaths from lung cancer is more than prostate, breast, and pancreatic. In 2022, there will be approximately 609,360 people diagnosed with lung cancers in the US, and there will be 1700 deaths due to lung cancer per day [2]. Overall, cancer symptoms are not easily found by doctors or patients without medical approaches such as magnetic resonance imaging (MRI), computed tomography (CT) scan, X-ray, molecular diagnosis, or ultrasound.

Using machine learning to analyse medical images by pattern recognition and classification is already widely used in computer-aided diagnosis [3]. However, the main challenge in this area is limited labelled data: First, labelling data is costly and time-consuming; Second, there are limited datasets in the biomedical domain. Hence, we proposed a hybrid framework called lung cancer GAN together with transfer learning (LCGANT) to solve these challenges. It combines a data augmentation (DA) technique called lung cancer deep convolutional GAN (LCGAN) to produce synthetic datasets of lung cancer images, and a regularisation enhanced transfer learning model called VGG-DF to classify different lung cancer classes.

We evaluate the effectiveness of our framework by comparing it with related methods for the same dataset. We explore whether the datasets generated by the LCGAN can efficiently improve the performance of lung cancer classification by VGG-DF and discuss the differences between the original dataset and the synthetic dataset. The main contributions of our work are as follows:

- A novel hybrid framework LCGANT is proposed to classify lung cancer images and solve the overfitting problem of lung cancer classification tasks.
- The proposed LCGANT framework is better than other state-of-the-art approaches.
- A lung cancer deep convolutional GAN (LCGAN) can generate synthetic lung cancer datasets to solve lung cancer classification tasks’ limited labelled data issue. A regularization enhanced model called VGG-DF can prevent overfitting problems with pre-trained model auto-selection.

## 2 Related Work

### 2.1 Cancer Classification

In 2020, Ref. [4] proposed a light fully automated deep learning model for cervix classification and detection. They reached 20 times faster than other state-of-art models, and the proposed pipeline is suitable for deployment on mobile devices.

Ref. [5] recommended an optimised InceptionV3 CNN architecture to classify four classes of lung cancer. They also analysed the underlying limitations of the existing CNN image classification for lung tumors.

Ref. [6] compared the different machine learning and deep learning models for colorectal cancer (CRC) lymph node metastasis (LNM) classifications. They investigated their experiments and found that the transfer learning methods reached an accuracy of 0.7583 and an area under the curve of 0.7941. The classification heat-map is used to improve the interpretability of results.

The optimal deep neural network (ODNN) and linear discriminate analysis (LDA) were designed by Ref. [7] in 2018 for lung cancer classification. Their results showed that the classifier resulted in an accuracy of 94.56%, specificity of 94.2%, and sensitivity of 96.2%.

Motivated by the above works, we decided to use pre-trained transfer learning models to evaluate the quality of images generated by the proposed LCGAN model for lung cancer classification.

### 2.2 Data Augmentation

It is common sense that the machine learning model’s performance will be better if there are more data that can be trained in the model [8]. Traditional transformations use geometric transformations to process the training data, such as flipping, random cropping, colour augmentation, and noise injection.

Apart from the traditional transformations, Ref. [9] demonstrated an augmentation method in the feature space that can be used to improve the performance of the supervised models. The technique can improve the changeability of the dataset and build a more robust model. Generative adversarial network (GAN) is another powerful DA technique proposed by Ref. [10] that includes two models: the generative and the discriminative models. The discriminator cannot distinguish whether the image generated by the generator is real or fake if the generator is training well. Since the concept of GANs was proposed, there have been different variants created such as deep convolutional GAN (DCGAN) [11], conditional GAN (cGAN) [12], cycle-consistent GAN (CycleGAN) [13], and stylebased GAN (styleGan) [14]. Adversarial training is also a potential solution for data augmentation, and it shows great success in improving the resolution of images [15]. Neural style transfer [16] manipulates sequential data by CNN to another style of the image, which preserves the features of the original image. It is well known for artistic applications. Moreover, the concept of meta-learning also has become very popular since Ref. [17] suggested it.

There is also some related work from the field of science such as [18,19]. These related studies provide the scientific background of this study.

### 2.3 Pre-Trained CNN Architecture for Feature Extraction

#### 2.3.1 VGG-16

The VGG-16 [20] increases the depth of the model by using small 3 × 3 convolution filters in the layers and shows excellent performance even for the small dataset. The VGG-16 was trained on the ImageNet dataset [21], which has over 14 million images for 1000 classes. The VGG-16 achieved an accuracy of 92.7%. It became one of the most efficient models in the ImageNet Large Scale Visual Recognition Challenge (ILSCRC) in 2014.

#### 2.3.2 ResNet50

ResNet50 [22] is a residual learning framework with 50 layers. Normally, neural networks are difficult to train when they have deeper layers. The ResNet50 reconstructs layers as learning residual functions and connects to the input layer of each residual block. The residual block skips the connection between different layers to solve the exploding gradient problem. The ResNet50 was trained on the ImageNet dataset [21], and its pretrained network can classify 1000 different classes.

#### 2.3.3 DenseNet121

Each layer in the DenseNet121 [23] architecture will take all the feature maps of preceding layers as input, and the feature map of the current layer will be used as input to all subsequent layers. The layer in the DenseNet121 is different from the traditional layer. The traditional layer only connects with adjacent layers. This feature allows the network to have fewer parameters, and it is more efficient than traditional CNNs.

#### 2.3.4 EfficientNet

EfficientNet [24] is an efficient scaling method that can scale the image’s dimensions by an effective compound coefficient. The network carefully balances the relations between the depth, width, and resolution to improve performance. The effectiveness of EfficientNet was evaluated on the MobileNet and ResNet. There are also a series of models called EfficientNets, and EfficientNet-B7 achieves the 97.1% accuracy on ImageNet.

## 3 Materials and Methods

### 3.1 Dataset

**Figure 1:**
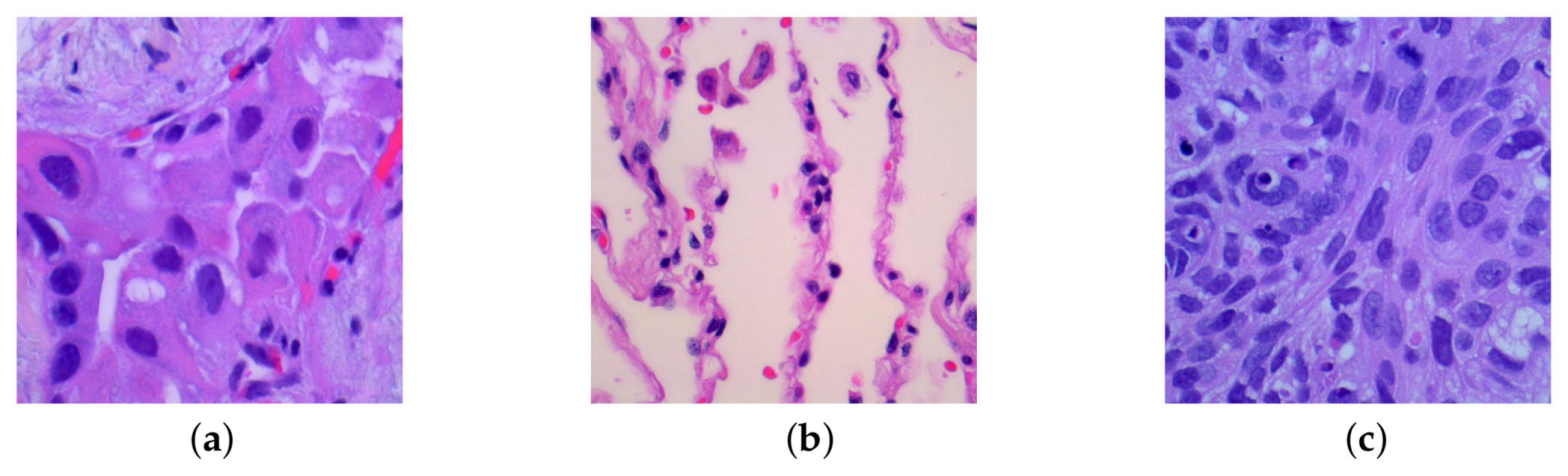
Three examples of different lung cancer classes from the original dataset with size 768 × 768: (**a**) lung adenocarcinoma, (**b**) lung benign, (**c**) lung squamous cell carcinoma.

We use a dataset that contains 15,000 histopathological images with three different lung cancer classes from [25]. All images are 768 × 768 pixels from the original 1024 × 768 pixels, and each class has 5000 images. The different classes of lung cancer in the dataset are shown as follows: lung adenocarcinoma is shown in Figure 1a; it is the most common cancer in the US, and it has a strong relationship with smoking habits; lung benign tissue is shown in Figure 1b; it is an abnormal tissue that is not cancerous; and lung squamous cell carcinoma is shown in Figure 1c; it always located in the central part of the lung or the main airway. All three examples are shown in Figure 1, and all the images are de-identified, HIPAA compliant, and validated sources.

### 3.2 Proposed LCGANT Framework for Lung Cancer Classification

Our framework contains three parts: we will illustrate the first part—LCGAN—in Section 3.2.1 and discuss the second part—regularization enhanced transfer learning model VGG-DF—in Section 3.2.2. The last part in Section 3.2.3 describes the model auto-selection method.

#### 3.2.1 Image Synthesis Using LCGAN

Our dataset pre-processing of LCGAN includes two steps: First, we use images of each class as a single dataset to train three LCGAN models individually. Second, we use three trained LCGAN to generate different synthetic images for each class. The original dataset is resized as 64 × 64 dimensions with 256 batch size, and we also use bilinear interpolation as an interpolation algorithm for resizing. A bilinear interpolation algorithm can avoid generating strange checkerboard patterns of images. After the training process of LCGAN is finished, we generate 10,000 synthetic images for each class of lung cancer with size 64 × 64; we then have two datasets after the LCGAN training process is finished. One is the original dataset with size 768 × 768; the other one is generated by the LCGAN with size 64 × 64. Before we use these datasets to train transfer learning models, we construct two new datasets: the real dataset with size 64 × 64, which is resized from the original dataset (15,000 images in total, each class has 5000 images); and the real and synthetic dataset with size 64 × 64, which combines the original dataset and synthetic dataset (45,000 images in total, 15,000 images for each class, the images in each class includes 5000 real images and 10,000 synthetic images). We then split the training and test datasets in 70:30 ratio for both datasets.

The idea of LCGAN is inspired by the deep convolutional generative adversarial networks (DCGANs) [11]. It is a class of unsupervised learning CNNs and contains traditional convolutional architecture with certain architecture constraints. Nonetheless, the basic theory for both LCGAN and DCGANs is from the GANs [10]. The whole GANs contain two parts: a generative model G called generator, which can capture the data distribution to manufacture outputs; and a discriminative model D called discriminator, which can estimate the outputs from the generator whether they are artificially created. During the training procedure of the generator, it will improve the chance for the discriminator to make mistakes. The idea was inspired by the famous strategy of the minimax two-player game. If the probability of real and fake images are both equal to $\frac{1}{2}$ for the discriminator, we can say that the generator has enough abilities to generate artificial images. The mathematical representation of this theory will be explained in the next paragraph.

We assume *p_data_* is the generator’s distribution over data x. The mapping from input noise variables *p_z_*(*z*) to data space is defined as *G* (z, *θg*). The *G* is regarded as the generator with parameters *θg*. Similarly, *D(x, θd)* is a discriminator with parameters *θg* that can take the real data or data from the generator to output a single scalar called *D* (*x*). During the training process, we train discriminator *D* to increase the accuracy of classifying the real images. Simultaneously, we train generator *G* to generate fake images close to real images during the training process. Overall, the formula tries to train a discriminator that can maximise the probability of distinguishing real images and synthetic images. It also trains a generator that can minimise the probability of distinguishing real and fake images by the discriminator. Finally, the generator can generate synthetic images that look like real images, and the discriminator cannot find the differences between them. The formula can be described as a function *V*(G, D) below:

(1) $$\begin{array}{ll} \underset{G}{\text{min}}\;\underset{D}{\text{max}}\;V(D,G) & ={𝔼}_{x\sim p_{\text{data}}(x)}[\text{log}D(x)]\\ & +{𝔼}_{z\sim p_{z}(z)}[\text{log}(1-D(G(z)))]. \end{array}$$

The LCGAN inherits some default settings from the DCGANs [11]:

- Replace the fully connected layers with a uniform noise distribution for the generator;
- Use the sigmoid function of the flattened layer for the discriminator;
- Add a Batch Normalization [26] layer to generator and discriminator to avoid poor initialization problems during the training process. The algorithm of Batch Normalization is shown in Equation (2). Here, we use *B* to represent a mini-batch of an entire training set with *m* examples. Then we can calculate the mean and variance of the mini-batch. Subsequently, we normalize the data *x_i_* in the mini-batch. The *e* is an arbitrarily small constant for numerical stability. Finally, we implement a transformation step to scale and shift the output.
- All layers in the discriminator use the LeakyReLU function.

(2) $$\begin{array}{ll} \mu_{Β}\leftarrow \frac{1}{m}\sum_{i=1}^{m}x_{i} & \text{mean}\;\text{of}\;\text{mini-batch,}\\ \sigma_{Β}^{2}\leftarrow \frac{1}{m}\sum_{i=1}^{m}{\left(x_{i}-\mu_{Β}\right)}^{2} & \text{variance}\;\text{of}\;\text{mini-batch,}\\ {\hat{x}}_{i}\leftarrow \frac{x_{i}-\mu_{Β}}{\sqrt{\sigma_{Β}^{2}+\in }} & \text{normalize,}\\ y_{i}\leftarrow \gamma {\hat{x}}_{i}+\beta \equiv {\text{BN}}_{\gamma ,\beta }\left(x_{i}\right) & \text{scale}\;\text{and}\;\text{shift}. \end{array}$$

**Figure 2:**
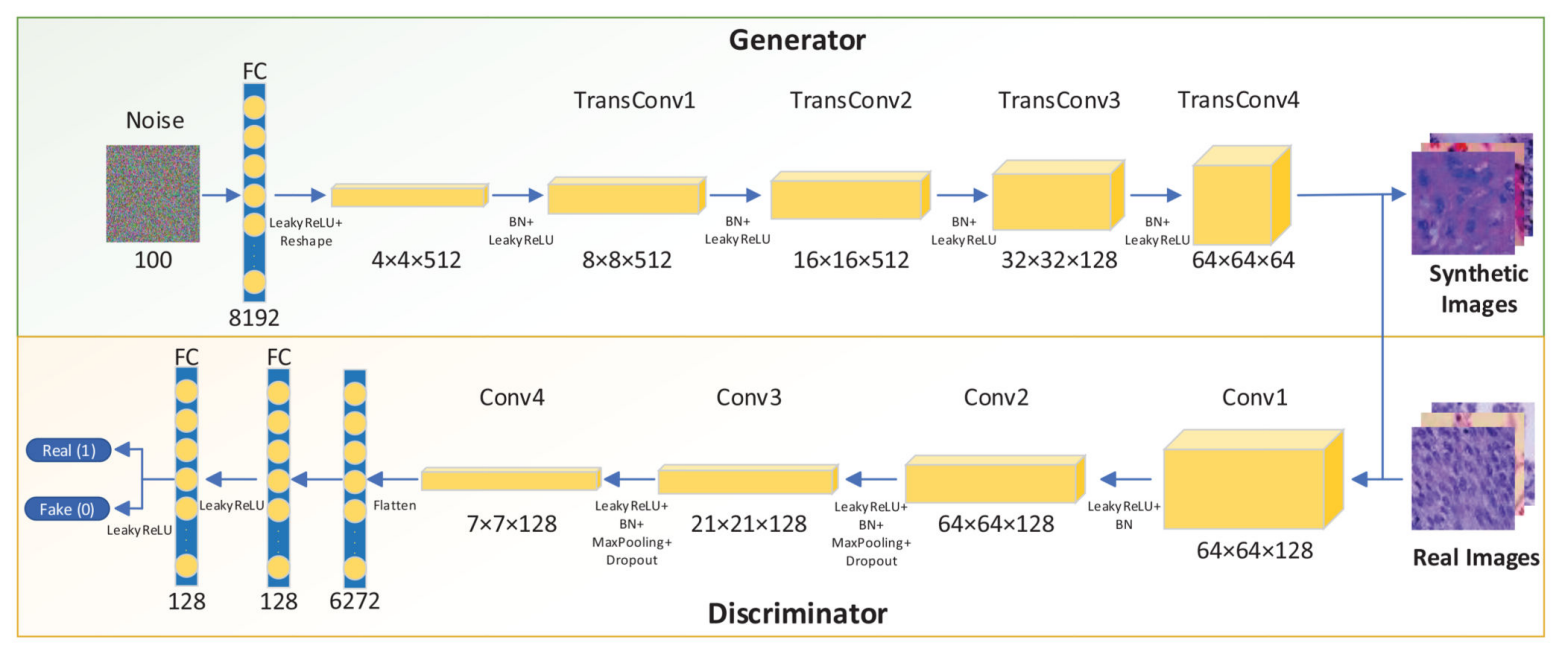
The overall architecture of proposed LCGAN.

Initially, we used most of the default settings from the DCGANs to train our models. The generated images always blur, and some checkerboard patterns are present in the synthetic images. Therefore, beyond the default setting from the DCGANs, we redesigned some features based on the default settings. The overall architecture of LCGAN is shown in Figure 2, and the innovations of LCGAN are shown as follows:

1. Try to add more filters in the front layers of the generator. More filters in the front layers can help the generator to get more activation maps to avoid missing essential features of the original image. Without sufficient filters, the generator will produce blurry images.
2. Use the LeakyReLU activation function for all the layers in the generator except the output layer with the Tanh function.
3. Add several dropout layers in the discriminator to avoid overfitting.
4. Avoid checkerboard artefacts:
  - During image pre-processing, we use the bilinear interpolation algorithm when we resize the original size to 64 × 64. The bilinear interpolation algorithm is used to do two-dimensional interpolation for a rectangle. First, we find four points of a rectangle: (*x*_1_, *y*_1_), (*x*_1_, *y*_2_), (*x*_2_, *y*_1_), and (*x*_2_, *y*_2_). Second, we assume the values of four points are *Q*_11_ for (*x*_1_, *y*_1_), *Q*_21_ for (*x*_1_, *y*_2_), *Q*_12_ for (*x*_2_, *y*_1_), and *Q*_22_ for (*x*_2_, *y*_2_). Finally, we can estimate the value of the formula at any point (*x, y*). The algorithm can refer to Equation (3).
  - Inspired by the idea from [27], the author uses sub-pixel convolution to get a better performance in image super-resolution. Set the kernel size that can be divided by the stride, and try to make the kernel size as big as possible.

(3) $$\begin{array}{ll} f(x,y)\approx & \frac{f\left(Q_{11}\right)}{\left(x_{2}-x\right)\left(y_{2}-y_{1}\right)}\left(x_{2}-x\right)\left(y_{2}-y\right)\\ & +\frac{f\left(Q_{21}\right)}{\left(x_{2}-x_{1}\right)\left(y_{2}-y_{1}\right)}\left(x-x_{1}\right)\left(y_{2}-y\right)\\ & +\frac{f\left(Q_{12}\right)}{\left(x_{2}-x_{1}\right)\left(y_{2}-y_{1}\right)}\left(x_{2}-x\right)\left(y-y_{1}\right)\\ & +\frac{f\left(Q_{22}\right)}{\left(x_{2}-x_{1}\right)\left(y_{2}-y_{1}\right)}\left(x-x_{1}\right)\left(y-y_{1}\right). \end{array}$$

**Figure 3:**
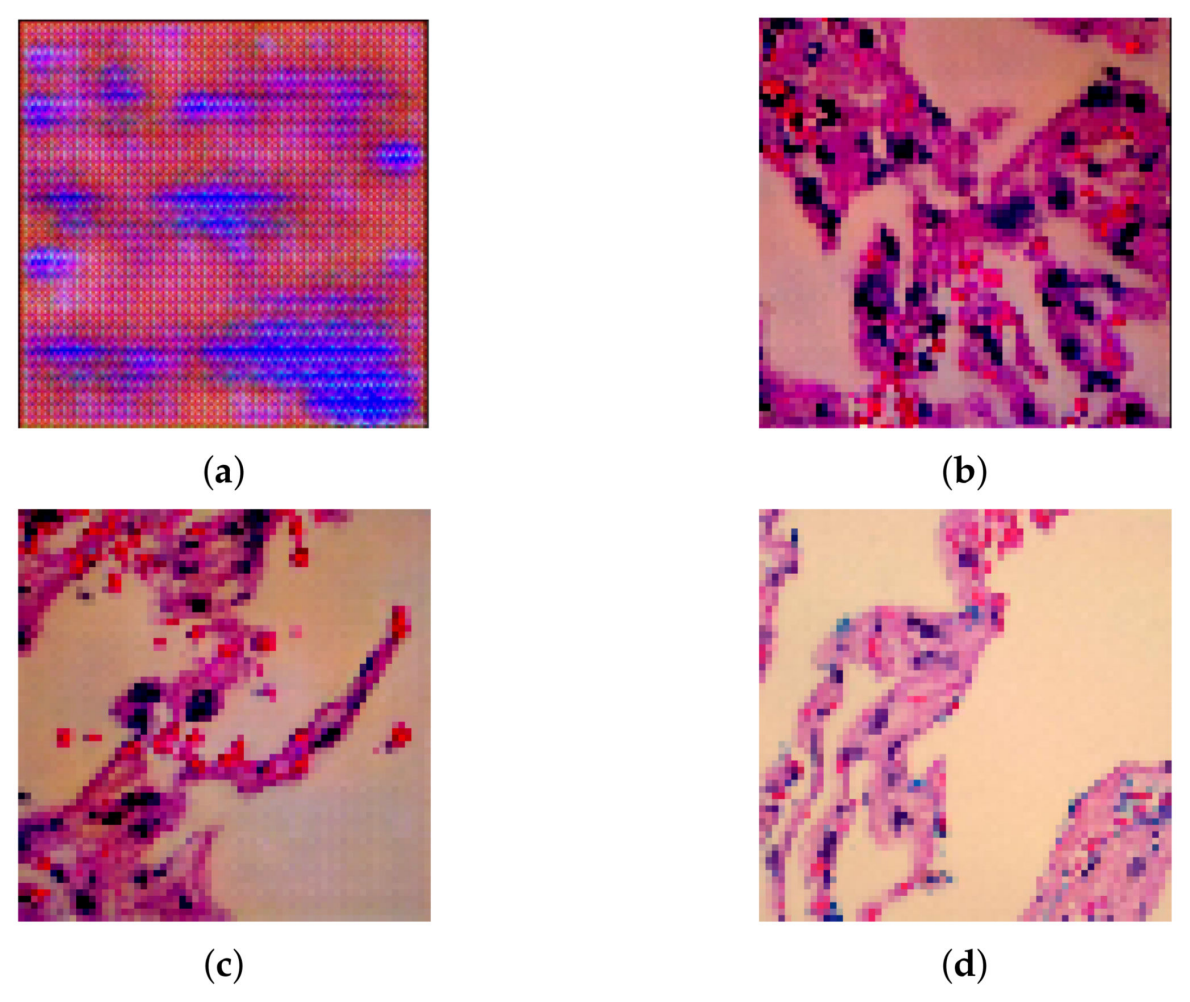
Synthetic lung benign images generated from the different version of LCGAN: (**a**) image from the initial version of LCGAN, (**b**) image from LCGAN which applies avoiding checkerboard methods, (**c**) image from LCGAN which applies all the proposed settings, (**d**) image from original dataset.

Figure 3 shows synthetic lung benign images based on the different versions of LCGAN. As Figure 3a shows, the initial version of LCGAN uses the default settings of the DCGANs, and it causes checkerboard patterns and blurry images. After using our settings, Figure 3b does not have a checkerboard pattern anymore, but it is still blurry. After applying all of the methods, Figure 3c becomes clearer than Figure 3b. By comparing with the real image shown in Figure 3d, the LCGAN generates clearer images that are difficult to distinguish from the real images.

#### 3.2.2 Regularization Enhanced Transfer Learning Model

The second part of the LCGANT framework is based on the pre-trained transfer learning models. We use four different pre-trained models in our framework: VGG-16, ResNet50, DenseNet121, and EfficientNetB4. Beyond the traditional transfer learning process, we add the dropout and fine-tuning techniques to prevent the overfitting problem and improve the classification performance. Finally, we found that our modified VGG-16 model got the best performance, and we call it VGG-DF.

**Figure 4:**
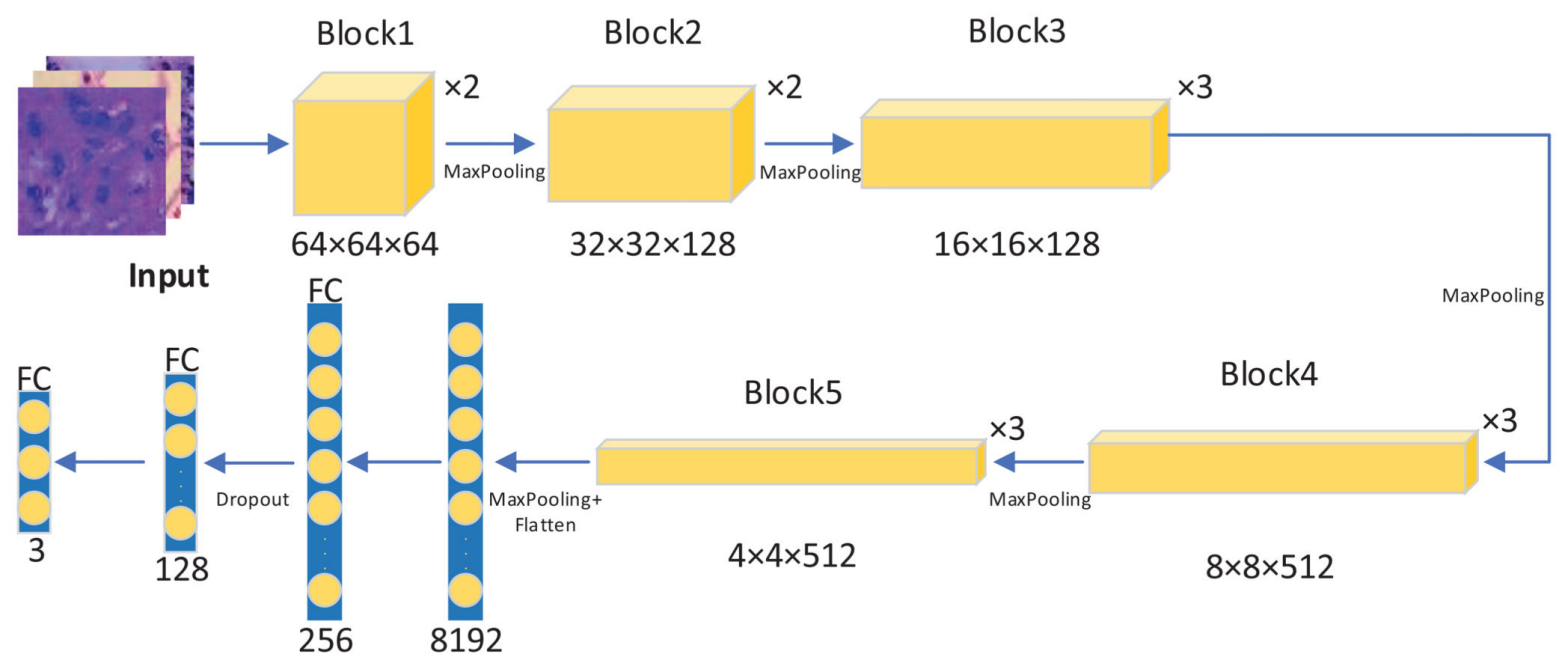
The overall architecture of the proposed VGG-DF.

The overall architecture of VGG-DF is shown in Figure 4. The entire architecture of VGG-DF includes five blocks, and there are different layers in each block.

The large dataset requires sufficient computational power and time to train a model with better performance, and it is not always ideal to train a model with a large dataset from scratch. However, if a model is only suitable for specific data and cannot efficiently work on different datasets, the robustness of the model is poor, and it cannot be widely used in different domains. Fortunately, the concept of transfer learning can easily use pre-trained models to adapt to similar datasets.

The typical workflow of transfer learning starts with taking layers of the pre-trained model. We load the four different models in our proposed framework: VGG-16, ResNet50, DenseNet121, and EfficientNetB4. The second step is to freeze all the pre-trained model layers to preserve the existing parameters. We then add trainable layers on top of the pre-trained models. These layers will be trained to fit the new dataset. In all the pre-trained models, the last layer is configured with a softmax function to classify three classes of lung cancer. The fourth step is training the added layer with the dataset. We also use fine-tuning to unfreeze the entire model, and re-training the entire model with a low learning rate can help the entire model adapt to the dataset.

To prevent the overfitting problem, we add a dropout layer to each pre-trained model to improve the generalization ability of the classification performance. Deep learning neural networks are likely to produce the overfitting problem when the neural network model has plenty of parameters with an insufficient dataset. On the one hand, the inadequate performance of the model does not have enough ability to extract the feature maps of the dataset; On the other hand, if the model fits the training dataset too well, it causes the overfitting problem. The overfitting problem typically means that the model has good performance with the training data, but it does not generalize well on the test dataset.

When training each batch of the dataset, the dropout layer randomly removes the neurons according to the given dropout probability of 0.2, and only the weights of retained neurons are updated. Due to the random elimination of neurons, the sparsity of the model improves to a certain level, which can efficiently reduce the synergistic effect. It also weakens the joint adaptability between neurons and finally enhances the generalization ability and robustness of the model. The algorithm of dropout for each neuron shows in Equation (4). In this equation, each intermediate activation *a_i_* is replaced by a value $a_{i}^{\prime }$ with a dropout probability *p*.

(4) $$a_{i}^{\prime }=\{\begin{array}{ll} 0 & \text{with}\;\text{probability}\;p\text{,}\\ \frac{a_{i}}{1-p} & \text{otherwise}. \end{array}$$

All four of these transfer learning models are first trained on the original dataset and we record the results. The original dataset has 15,000 images that belong to three classes of lung cancer. After training on the original dataset, LCGAN generates 30,000 images, and each class has 10,000 new images. We then record the results for it. We will evaluate the results in Section 4.

#### 3.2.3 Pre-Trained Model Auto-Selection

To ensure that we can get the best performance of each transfer learning model during the training, we use two callback functions to save the training time and the best model during the training process. The functions are model checkpoint callback and early stopping callback. After we get the model, we automatically compare the performance of each model and choose the model with the best performance of the four pre-trained models.

The model checkpoint callback function will monitor the overall performance of the current training model. It will save the model when the current model gets the best performance. As a result, the final model will have the best performance within all the model variants during the training process. An early stop callback function is also used in our framework. The early stop callback function will monitor the validation loss with the patience of 10 epochs, then try to stop the training process when there are no improvements within 10 epochs.

When we finished the training process of four transfer learning models, we compared the performance of each model and chose the VGG-DF with the best performance as the second part of the LCGANT framework.

## 4 Results

### 4.1 Set-Up of Experiments

The hyperparameters of the experiments are shown in Table 1. The LCGAN and VGG-DF are running on a TESLA P100 16GB RAM GPU provided by the Kaggle service, as well as the Intel Xeon CPU with 13GB RAM. The data augmentation for the original dataset to the new dataset with 64 × 64 dimensions was trained on a GTX 1070 8GB GPU with i7-8700k CPU and 32GB RAM.

The detailed structures of the generator and discriminator are shown in Tables 2 and 3, respectively.

### 4.2 Results and Analysis

#### 4.2.1 Lung Images Generated by DCGAN Generator

**Figure 5:**
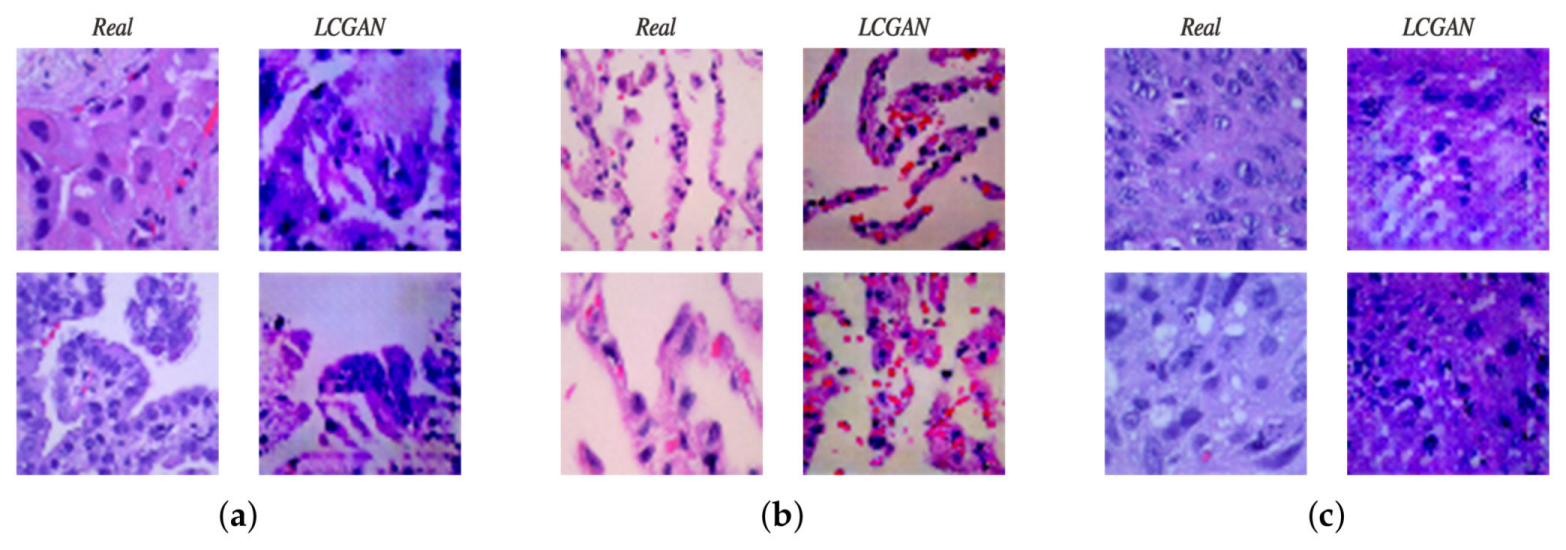
Comparison between real and LCGAN images: (**a**) lung adenocarcinoma, (**b**) lung benign, (**c**) lung squamous cell carcinoma.

Figure 5a illustrates examples of lung adenocarcinoma images. The two images on the left side are real, and the two images on the right side are synthetic images from the LCGAN. As we can see, the synthetic images look very similar to the real images, and synthetic images also inherit the patterns and features from the original lung cancer images. Additionally, for the lung benign tissue images shown in Figure 5b and the lung squamous cell carcinoma images shown in Figure 5c, the left sides of these figures are real images, and the right sides are synthetic images. Although there are some slight differences between real images and synthetic images, they do not affect the performance improvement. After generating synthetic images, we do experiments based on different transfer learning models to evaluate the performance of our LCGAN model. The results are discussed in the following Section 4.2.2.

#### 4.2.2 the Results of Different Transfer Learning Models with Different Training Datasets

We run two experiments based on different datasets. Each experiment trains four different transfer learning models, including VGG-DF, ResNet50, DenseNet121, and EfficientNetB4. The first experiment was trained on the original dataset, and the second experiment was trained on the dataset that combines the original and synthetic datasets generated by LCGAN. First, we compared results between different transfer learning models with the same dataset so we can find the most efficient model by this comparison. Second, we compared the results between different datasets with the same model so we can evaluate whether the synthetic dataset improves the performance of same transfer learning model. We ran both experiments 10 times and calculated the average metrics for each model.

As Table 4 shows, the VGG-DF, ResNet50, DenseNet121, and EfficientNetB4 reach average classification accuracy of 95.80%, 95.56%, 53.87%, and 45.96%, respectively. We can see that the VGG-DF shows the highest accuracy of 95.80%, precision of 95.81%, sensitivity of 95.80%, and F1-score of 95.80%, and the EfficientNetB4 shows the lowest performance. ResNet50 also shows good performance, close to the results of VGG-DF. Overall, the first experiment shows that the VGG-DF and ResNet50 models perform better than the DenseNet121 and EfficientNetB4 models.

Table 5 shows the results of the second experiment in which training on the dataset combines the original and synthetic datasets. The four models reach classification accuracies of 99.84% for VGG-DF, 95.46% for ResNet50, 79.64% for DenseNet121, and 51.74% for EfficientNetB4. These are the same as for the first experiment. The VGG-DF model shows the best result with an accuracy of 99.84%, precision of 99.84%, sensitivity of 99.84%, and F1-score of 99.84%. The ResNet50 shows the nearest results with VGG-DF; the DenseNet121 and EfficientNetB4 show 79.64% and 51.74% for accuracy, respectively. We can conclude that the VGG-DF and ResNet50 models show the best performance for the lung cancer classification task. The DenseNet121 and EfficientNetB4 did not work very well compared with the other models in the second experiment.

To evaluate the quality of synthetic images generated by the LCGAN, we compare the results from Tables 4 and 5. As we noted previously, Table 4 shows the first experiment’s results, for which all the training images are real images. Table 5 shows the results of the second experiment, for which the training dataset combines original images and synthetic images. First, we compare the VGG-DF model from the two tables. Accuracy, precision, sensitivity, and F1-score improve by 4.04%, 4.03%, 4.04%, and 4.04% respectively, with extra synthetic data. The ResNet50 model improves by 3.90%, 3.93%, 3.90%, and 3.90% for each metric with extra synthetic data. For the third model, the accuracy of DenseNet121 increases by 25.77%, the precision decreases by 10.05%, sensitivity increases by 5.32%, and the F1-score improves by 2.44%. The last model is EfficientNetB4, for which the accuracy, sensitivity, and F1-score increased by 5.78%, 5.78%, and 4.59%, respectively, and the precision decreased by 7.23%. To summarize these comparisons, the synthetic data generated by the LCGAN remarkably improve the lung cancer classification performance for the VGG-DF and ResNet50 models. For DenseNet121 and EfficientNetB4, the accuracy, sensitivity, and F1-score are increased, whereas the precision is not. Especially for the VGG-DF and ResNet50, the overall performance is very good.

### 4.3 Comparison with State-Of-Art Methods for the Same Dataset

In Table 6, we compared our outcome with other related work for the same dataset. Most related work uses the CNN model to classify lung cancers, and there is no one using the GANs to improve the performance of the models. Our framework is the only one that combines the GANs and CNN models. By comparing the matrix with other work, our framework reached the best accuracy, precision, sensitivity, and F1-score. Furthermore, we believe that our framework is also suitable for other datasets to improve performance by using additional synthetic data.

## 5 Discussion

This study proposed the LCGANT, which includes a lung cancer deep convolutional GAN called LCGAN with regularization enhanced transfer learning model VGG-DF. TheLCGAN can generate additional synthetic lung cancer images, and the VGG-DF can use original and synthetic datasets to classify different lung cancer classes. Our results indicate that the synthetic lung cancer images are indistinguishable from the original images, and the synthetic images indeed can improve the performance of backbone models.

Generative adversarial networks (GANs) are one of the unsupervised deep learning models used in medical image analysis [34]. There are also other models like autoencoders (AEs) and variants, restricted Boltzmann machines (RBMs), deep belief networks (DBNs), and deep Bolzmann machines (DBMs). Recently, GANs have made great progress in different domains. In our proposed LCGAN, we add extra settings to help the generator produce high-quality lung cancer images and use different techniques to avoid the checkerboard effects of generated images.

After we completed the training process of the LCGAN, we add synthetic images as an additional dataset to train our regularization enhanced VGG-DF model. As Section 4 describes, the synthetic images improve the performance of VGG-DF. It also proves that the synthetic images generated by our LCGAN can be applied to train deep learning models that resolve the limit labelled lung cancer images in the biomedical domain. However, the validity of synthetic images generated by the LCGAN for diagnostic applications has not been provided. Therefore it has the potential risk of making conclusions for clinical or scientific applications.

This study has several limitations. Alhough the proposed framework got good results, there are still some drawbacks to the current framework:

- The synthetic images generated by the LCGAN have slight differences from real images.
- The dimension of images produced by the generator is 64 × 64. This is not sufficient for the biomedical domain. Images with high resolution are very essential in this area.

In our future work, we will evaluate the current framework on the different datasets and explore other potential GANs in the biomedical domain. Moreover, we will explore the reasons for the differences between real and synthetic data and design a more effective architecture to create high-resolution synthetic images.

## 6 Conclusions

This article proposed a framework LCGANT for addressing the overfitting problem of lung cancer classification tasks. The framework contains two main parts: the lung cancer deep convolutional GAN (LCGAN) to generate synthetic lung cancer images and the regularization enhanced transfer learning model VGG-DF to classify lung cancer datasets. The dataset of VGG-DF contains original and synthetic images. The result shows that the performance of different classification models improved by adding additional synthetic data from the LCGAN. Based on the results in Tables 4 and 5, our LCGANT showed the best performance with the results of: 99.84% (accuracy), 99.84% (precision), 99.84% (sensitivity), and 99.84% (F1-score). The results prove that the proposed LCGANT is an effective framework for solving the overfitting problem of lung cancer classification tasks. It also achieves better performance compared with other state-of-art methods.

## Figures and Tables

**Table 1:** Hyperparameter settings of LCGAN.

Hyperparameter	Value
Optimizer	Adam
Loss (LCGAN)	Binary Crossentropy
Loss (VGG-DF)	Categorical Crossentropy
Dropout Probability	0.2
Batch Size	256
Number of Epochs (LCGAN)	15,000
Number of Epochs (VGG-DF)	5
Kernel Size (G)	4
Kernel Size (D)	3

**Table 2:** The architecture of the generator.

Layer (Type)	Output Shape	Number of Parameters
Input	100	0
Dense	8192	827,392
LeakyReLU	8192	0
Reshape	4 × 4 × 512	0
TransConv	8 × 8 × 512	4,194,816
BatchNorm	8 × 8 × 512	2048
LeakyReLU	8 × 8 × 512	0
TransConv	16 × 16 × 512	2,097,408
BatchNorm	16 × 16 × 512	1024
LeakyReLU	16 × 16 × 512	0
TransConv	32 × 32 × 128	524,416
BatchNorm	32 × 32 × 128	512
LeakyReLU	32 × 32 × 128	0
TransConv	64 × 64 × 64	131,136
BatchNorm	64 × 64 × 64	256
LeakyReLU	64 × 64 × 64	0
TransConv	64 × 64 × 3	3075
Activation	64 × 64 × 3	0
Total Parameters: 7,782,083		
Trainable Parameters: 7,780,163		
Non-trainable Parameters: 1920		

**Table 3:** The architecture of the discriminator.

Layer (Type)	Output Shape	Number of Parameters
Input	64 × 64 × 3 0	
Conv2D	64 × 64 × 128	3584
LeakyReLU	64 × 64 × 128	0
BatchNorm	64 × 64 × 128	512
Conv2D	64 × 64 × 128	147,584
LeakyReLU	64 × 64 × 128	0
BatchNorm	64 × 64 × 128	512
MaxPooling	21 × 21 × 128	0
Dropout	21 × 21 × 128	0
Conv2D	21 × 21 × 128	147,584
LeakyReLU	21 × 21 × 128	0
BatchNorm	21 × 21 × 128	512
Conv2D	21 × 21 × 128	147,584
LeakyReLU	21 × 21 × 128	0
BatchNorm	21 × 21 × 128	512
MaxPooling	7 × 7 × 128	0
Dropout	7 × 7 × 128	0
Flatten	6272	0
Dense	128	802,944
LeakyReLU	128	0
Dense	128	16,512
LeakyReLU	128	0
Dense	1	129
Total Parameters: 1,267,969		
Trainable Parameters: 1,266,945		
Non-trainable Parameters: 1024		

**Table 4:** Results of different transfer learning models trained on the original dataset.

	VGG-DF (Ours)	ResNet50	DenseNet121	EfficientNetB4
Accuracy	95.80%	95.56%	53.87%	45.96%
Precision	95.81%	95.56%	76.86%	75.93%
Sensitivity	95.80%	95.56%	53.87%	45.96%
F1-score	95.80%	95.56%	49.62%	38.44%

**Table 5:** Results of different transfer learning models trained on the dataset that combines original and synthetic datasets.

	VGG-DF (Ours)	ResNet50	DenseNet121	EfficientNetB4
Accuracy	99.84%	99.46%	79.64%	51.74%
Precision	99.84%	99.49%	66.81%	68.70%
Sensitivity	99.84%	99.46%	59.19%	51.74%
F1-score	99.84%	99.46%	52.06%	43.03%

**Table 6:** Comparison of state-of-art methods.

Reference	Model (Method)	Accuracy	Precision	Sensitivity	F1-Score
[28]	RESNET50	93.91%	95.74%	81.82%	96.26%
	RESNET18	93.04%	96.81%	84.21%	95.79%
	RESNET34	93.04%	95.74%	80.95%	95.74%
[29]	Ensemble	91%	92%	91%	91%
	ResNet50V2	90%	91%	90%	90%
[30]	CNN-D	94.6%	-	-	-
[31]	DL-based CNN	96.33%	96.39%	96.37%	96.38%
[32]	CNN	97.2%	97.33%	97.33%	97.33%
[33]	XGBoost	99.53%	99.33%	99.33%	99.33%
Our method	**LCGANT**	**99.84%**	**99.84%**	**99.84%**	**99.84%**

## Data Availability

Data available in a publicly accessible repository that does not issue DOIs. Publicly available datasets were analyzed in this study. These data can be found here: https://github.com/tampapath/lung_colon_image_set (accessed on 6 April 2022).
